# Dynamics of molecular evolution in RNA virus populations depend on sudden versus gradual environmental change

**DOI:** 10.1111/evo.13193

**Published:** 2017-02-14

**Authors:** Valerie J. Morley, Paul E. Turner

**Affiliations:** ^1^ Department of Ecology and Evolutionary BiologyYale UniversityP. O. Box 208106New HavenConnecticut06520; ^2^ Graduate Program in MicrobiologyYale School of MedicineNew HavenConnecticut06520

**Keywords:** Adaptation, clonal interference, epistasis, experimental evolution, Sindbis virus

## Abstract

Understanding the dynamics of molecular adaptation is a fundamental goal of evolutionary biology. While adaptation to constant environments has been well characterized, the effects of environmental complexity remain seldom studied. One simple but understudied factor is the rate of environmental change. Here we used experimental evolution with RNA viruses to investigate whether evolutionary dynamics varied based on the rate of environmental turnover. We used whole‐genome next‐generation sequencing to characterize evolutionary dynamics in virus populations adapting to a sudden versus gradual shift onto a novel host cell type. In support of theoretical models, we found that when populations evolved in response to a sudden environmental change, mutations of large beneficial effect tended to fix early, followed by mutations of smaller beneficial effect; as predicted, this pattern broke down in response to a gradual environmental change. Early mutational steps were highly parallel across replicate populations in both treatments. The fixation of single mutations was less common than sweeps of associated “cohorts” of mutations, and this pattern intensified when the environment changed gradually. Additionally, clonal interference appeared stronger in response to a gradual change. Our results suggest that the rate of environmental change is an important determinant of evolutionary dynamics in asexual populations.

Populations can adapt by fixing a series of beneficial mutations that improve fitness in the selective environment. Characterizing these molecular dynamics quantitatively is a key step toward predicting how natural populations will respond to changes in their environments. Additionally, understanding evolutionary dynamics in asexual populations is of biomedical interest because evolving asexual lineages of tumor cells and infectious microbial pathogens cause millions of human deaths annually (Lozano et al. 2012). Here we focus on RNA viruses, whose potential for rapid evolution when adapting to novel hosts makes them particularly important as emerging pathogens (Cleaveland et al. 2001; Taylor et al. 2001; Woolhouse and Gowtage‐Sequeria 2005; Jones et al. 2008). Improved understanding of RNA virus evolution in response to changes in host community composition could help refine predictions on the likelihood of virus emergence success on new hosts such as humans.

In small asexual populations with low mutation rates, the dynamics of adaptation are relatively simple. Beneficial mutations are rare, so any beneficial mutation that escapes being lost to genetic drift sweeps to fixation (Haldane 1927; Gillespie 1984; Orr 2003; Good et al. 2012). However, if population size is large or mutation rate is high, the dynamics of adaptation become complicated by interference between mutations. Clonal interference occurs when multiple beneficial mutations are present simultaneously on different genetic backgrounds in a population. Because these mutations cannot be recombined onto the same background, beneficial mutations can be lost when one lineage outcompetes another (Gerrish and Lenski 1998; Wilke 2004). Additionally, multiple beneficial mutations can occur on the same genetic background before a lineage fixes. This creates a dynamic wherein only beneficial mutations that occur on the fittest possible backgrounds rise to fixation, and it results in clusters of mutations fixing simultaneously (Desai and Fisher 2007; Park and Krug 2007; Good et al. 2012). Interference dynamics in asexual populations depend on biotic factors such as population size, mutation rate, and the underlying distribution of mutational effects (Desai and Fisher 2007; Schiffels et al. 2011; Good et al. 2012). In the current study, we experimentally explore how these dynamics may also be influenced by the rate of environmental change.

The study of evolutionary dynamics has been revolutionized by next‐generation sequencing, which allows the frequency of alleles or lineages to be tracked over evolutionary time in experimental microbial populations (Barrick and Lenski 2009; Lang et al. 2013; Acevedo et al. 2014; Borderia et al. 2015; Levy et al. 2015; McDonald et al. 2016; Tenaillon et al. 2016) as well as in natural populations (Zanini et al. 2015). However, the majority of work on adaptation, both theoretical and empirical, considers a population that has experienced a sudden environmental change and then adapts to a novel but constant environment (Elena and Lenski 2003; Elena and Sanjuan 2007; Collins 2011). While sudden environmental changes do happen in nature, many environmental changes happen more gradually. The rate of environmental turnover is predicted to affect the dynamics of molecular adaptation; it may affect the dynamics of clonal interference, the number of mutations that fix simultaneously, and the order in which mutations of different effect sizes sweep to fixation (Bello and Waxman 2006; Collins et al. 2007; Kopp and Hermisson 2007; Collins and de Meaux 2009; Schiffels et al. 2011; Gorter et al. 2016). Here we examined Sindbis virus (SINV), the type species for positive‐sense ssRNA Alphaviruses, which include mosquito‐borne pathogens such as chikungunya virus and Eastern equine encephalitis virus. We used SINV evolution under sudden versus gradual host change as a model for studying the dynamics of adaptation in evolving asexual populations encountering different rates of environmental turnover.

Previously, we experimentally evolved SINV populations in replicated tissue‐culture environments to which we introduced a novel host cell type either suddenly or gradually (Morley et al. 2015). Our original laboratory host for SINV culture was BHK‐21, a mammalian‐derived cell line that is highly permissive for viral growth. In the experiment, SINV populations were transitioned onto the novel host cell type CHO pgsD‐667, a mammalian cell line deficient in its heparin sulfate synthesis pathway. This deficiency results in lower virus fitness on the novel host relative to the original host. BHK and CHO cells can be combined and co‐cultured, which permitted us to create mixed monolayers where we controlled the relative availability of the two host cell types in the environment. This technique allowed us to transition virus populations onto the novel host either suddenly or gradually. In the sudden treatment, SINV populations were evolved for 25 passages (∼100 virus generations) in replicated environments consisting solely of CHO cells. In the gradual treatment, SINV populations were passaged in mixed‐host environments that transitioned from being BHK cell dominated to CHO cell dominated, with the proportion of CHO cells in the environment increasing at each passage.

In the gradual treatment, viruses evolved in a heterogeneous host environment where the environmental composition changed directionally over time. The probability of encountering each cell type was expected to be proportional to the prevalence of that cell type. When populations evolve in spatially heterogeneous environments, the evolutionary outcome is expected to depend on the relative abundance and productivity of environmental patches, the amount of gene flow between patches, the heritability of niche width, and whether or not there is a performance trade‐off between patches (Roughgarden 1972; Kassen 2002; Jasmin and Kassen 2007; Morley et al. 2016). In our experimental system mutations can have different selection coefficients on the two cell types, and a mutation's selection coefficient in the mixed environment may change as the composition of the host community shifts. In our initial experiment we found that fitness gains on the two host types were positively correlated, but passage on the two host types selected for different molecular changes (Morley et al. 2015). Thus, we did not observe a SINV growth trade‐off on the two host types, but the selection coefficients of individual virus mutations on the two cell types may have varied in either magnitude or sign. SINV populations passaged in gradually changing environments more consistently reached high fitness on the novel host and were more likely to converge on the same molecular substitutions (Morley et al. 2015).

In the current study, we used next‐generation sequencing to analyze the dynamics of molecular evolution in SINV populations that experienced sudden versus gradual shifts in their host communities. To examine the dynamics of evolution over time, we sampled 18 virus populations (nine per treatment) at eight time points, or approximately every 12 generations of virus evolution. We conducted population‐level whole‐genome sequencing, which allowed us to track the frequencies of mutant alleles in the populations over the course of the experiment. To address the challenge of distinguishing sequencing errors from true genetic variants, each sample was amplified and sequenced in duplicate. This technical replication allowed us to dismiss variants that appeared in one but not both replicates as likely sequencing errors. While this method does not have the same power to accurately detect very low‐frequency variants compared to other methods (Acevedo et al. 2014; Kennedy et al. 2014), it was sufficient to analyze mutations that rose to appreciable frequencies and to track mutations that were detectable at multiple time points. Using this approach we mapped sequence evolution at a high temporal resolution across the entire genome.

Using these data, we evaluated three hypotheses. First, after sudden environmental change, beneficial mutations of large effect will fix early, followed by mutations of progressively smaller effect. In gradually changing environments, there will be no such correlation between mutational effect size and order of fixation. When a population adapts to a novel but constant environment, theory predicts that the beneficial alleles fixed will have exponentially distributed effect sizes, with a few large factors fixing early in the adaptive walk followed by a series of smaller factors (Orr 1998, 1999, 2002). This prediction has been supported by experimental work in evolving microbial populations (Holder and Bull 2001; Barrett et al. 2006; Betancourt 2009; Collins and de Meaux 2009) with some notable exceptions (Rokyta et al. 2005, 2008). On the other hand, populations in a gradually changing environment must track a moving fitness optimum, and the correlation between mutational effect size and time of fixation is predicted to be disrupted (Bello and Waxman 2006; Collins et al. 2007; Kopp and Hermisson 2007; Collins and de Meaux 2009).

Second, in both treatments, mutations will fix multiply more often than singly. If population size and mutation rate are suitably high, mutations may arise often enough to occur on the same genetic background and sweep to fixation together (Bollback and Huelsenbeck 2007). When populations are evolving with clonal interference, genomes containing multiple beneficial mutations are expected to outcompete genomes containing a single beneficial mutation, biasing fixation events toward clusters of linked mutations (Park and Krug 2007; Good et al. 2012). Alternatively, mutations may fix simultaneously due to genetic hitchhiking, which occurs when a beneficial mutation carries linked mutations to fixation (Smith and Haigh 1974; Gillespie 2000; Schiffels et al. 2011). Simultaneous fixation of cohorts of mutations has been observed in experimental and natural microbial systems (Strelkowa and Lässig 2012; Lang et al. 2013), and we would expect this dynamic in experimental RNA virus populations due to their high mutation rate.

Third, clonal interference will be stronger in the treatment with gradual environmental change. The dynamics of clonal interference may be influenced by environmental factors including harshness (Pepin and Wichman 2008), spatial heterogeneity (Campos et al. 2008), and the rate at which an environment changes (Schiffels et al. 2011). A slower environmental change results in weaker selective pressures early in adaptation. If weaker selective pressures result in slower selective sweeps, this may increase the likelihood of multiple beneficial mutations occurring simultaneously in the population and generating interference. Additionally, in a gradually changing environment the selective coefficients of alleles are time dependent. Schiffels et al. (2011) predicted stronger clonal interference when the selective coefficients of alleles change more frequently. Previous experimental evolution results were consistent with higher rates of clonal interference in more gradually changing environments, although the underlying genetics were not investigated (Collins and de Meaux 2009).

## Materials and Methods

### STUDY SYSTEM

SINV has an 11.7 kb unsegmented positive‐sense ssRNA genome containing nine genes that encode nonstructural proteins (nsP1, nsP2, nsP3, nsP4) and structural proteins (C, E3, 6K, E1, E2). SINV primarily reproduces asexually; however recombination between genomes co‐infecting the same cell is possible in positive‐sense RNA viruses. SINV recombination has been demonstrated in vitro; however most SINV recombination events are nonhomologous and only infrequently result in viable virus progeny (Weiss and Schlesinger 1991; Raju et al. 1995; Hajjou et al. 1996; Strauss and Strauss 1997). SINV samples examined in this study were produced as part of a previously published experiment. Methods for culture and experimental evolution were described previously (Morley et al. 2015). Briefly, virus populations were evolved for 25 passages in each treatment. At each passage, a cell monolayer was infected with approximately 10^4^ plaque‐forming units (pfu), giving a multiplicity of infection (MOI) of about 0.01 pfu per cell. Over the course of a 48‐h passage, virus populations grew to about 10^7^ pfu each. The composition of mixed cell monolayers is described in Figure S2. For the current study, 18 virus populations were selected for in depth analysis. Using a random number generator, we selected nine populations from the treatment with the most sudden change and nine from the treatment with the most gradual change.

### LIBRARY PREPARATION AND SEQUENCING

Genomic viral RNA was isolated from 144 virus samples (18 virus populations × 8 time points each) using the QIAamp Viral RNA mini kit (Qiagen). All subsequent steps, from reverse transcription through sequencing, were completed in duplicate, resulting in two technical replicates of each sample. cDNA was generated by reverse‐transcription with Superscript II (Life Technologies) using random hexamer primers. The entire genome sequence was amplified via PCR with GoTaq DNA Polymerase (Promega) using eight primer pairs, generating overlapping PCR fragments 1.5–2.1 kb in length. Amplified genome fragments were purified using the QIAquick PCR Purification Kit (Qiagen), and the eight fragments from each virus sample were combined to create an equimolar mixture. Libraries were prepared from the pooled viral amplicons using the Nextera XT Kit (Illumina Inc.) and the Nextera Index Kit (Illumina Inc.). Samples were sequenced in a single lane via paired‐end, 75‐bp read Illumina HiSeq 2500 (Illumina Inc.) at the Yale Center for Genome Analysis.

### GENOME ALIGNMENT AND VARIANT CALLING

Quality control of reads was conducted using Cutadapt version 1.8.3 with a minimum quality score of 20 and a minimum length of 30 bp (Martin 2011). Sequences were mapped to the consensus sequence of the ancestral virus used to found the evolution experiment using BWA version 0.7.10 (Li and Durbin 2009). Consensus sequences and minority variant tables were generated using QUASR version 7.01 (Watson et al. 2013) with a minimum (Watson et al. 2013) a minimum variant frequency cutoff of 0.01. Additional indels were called using VarScan version 2.3.9 (Koboldt et al. 2012). For each sample, comparing data from the two technical replicates generated a final variant table. Variants that appeared in only one technical replicate were assumed to be errors and were excluded. For variants detected in both technical replicates, the mean of the two replicate frequencies was included in the final variant table (see Figure S1 for analysis of error between technical replicates).

### COHORT ASSIGNMENT

Mutations were assigned to cohorts based on previously described methods (Lang et al. 2013). For this analysis, we excluded mutations that never reached a frequency of 30%. The frequency trajectory of each mutant allele was considered a vector in eight dimensions (frequencies at eight time points). The frequency vectors from each virus population were hierarchically clustered in R version 3.1.1 (R Development Core Team 2014), then hierarchies were flattened with a cutoff distance of 0.275. In each treatment, the number of mutations classified as singletons or members of a cohort were compared by two‐sided *t*‐test in R.

### ESTIMATION OF MUTATIONAL EFFECT SIZES

To estimate the relative fitness effect of beneficial mutations, we estimated *S_up_*: the fitness of genomes containing a given mutation relative to the mean fitness of the population when the mutation was first detected (Lang et al. 2011, 2013). For each mutation, we sought to identify *t_1_* and *t_2_*, the first consecutive time points such that the frequency of the mutation at *t_1_* was greater than zero and the frequency of the mutation at *t_2_* was greater than the frequency at *t_1_*. Only mutations that had a valid *t_1_* and *t_2_* based on these criteria were included in further analyses. For each mutation, we then calculated

Sup=1t2−t1 ln ft21−ft2− ln ft11−ft1,

where *f(t)* is the frequency of the mutation at time *t*. *S_up_* estimates the combined effects of the focal mutation and the genetic background it occurred on. For example, if a single genome that contains two mutations, one neutral and one beneficial, is rising in frequency, we would detect the same *S_up_* for both the neutral and the beneficial mutation. Three mutations in the dataset (two observed in the sudden treatment, and one in the gradual treatment) had already reached 100% frequency by *t_2_*. We were unable to calculate a valid *S_up_* value for these three mutations, so they were excluded. All plots were created using the packages ggplot2 and cowplot in R version 3.1.1 (Wickham 2009; R Development Core Team 2014; Wilke 2016).

## Results

### SEQUENCING ALLOWS HIGH‐RESOLUTION TRACKING OF ALLELE FREQUENCIES OVER TIME

**Figure 1:**
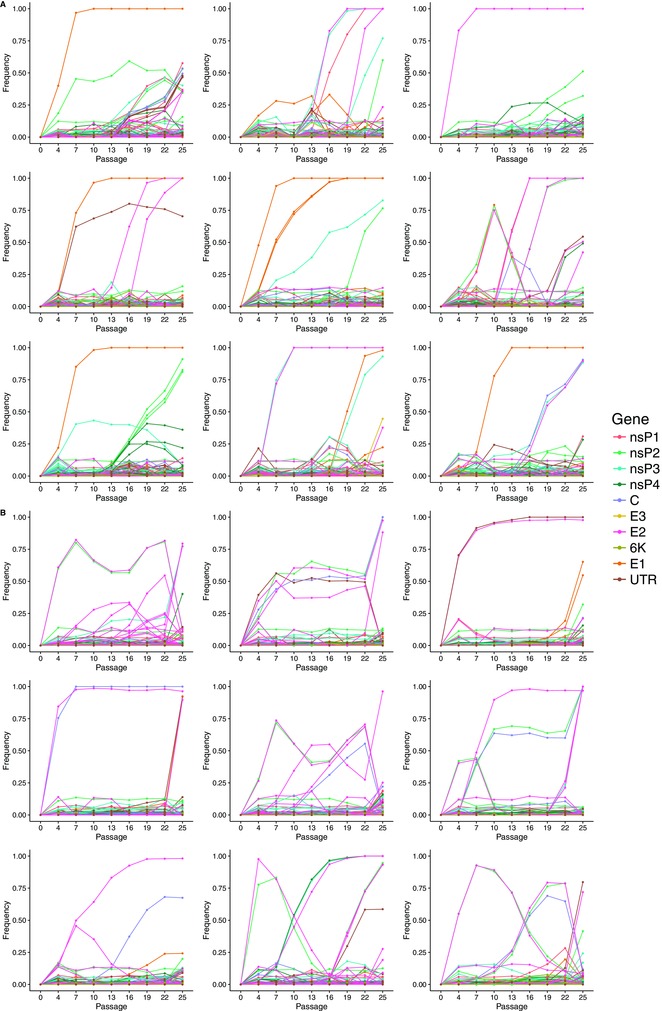
Trajectory plots showing the frequency of mutations relative to the ancestral sequence over time. Each plot shows dynamics in a single virus population over the course of the experiment (nine populations per treatment). Each line tracks the frequency of one mutation at each passage. Colors indicate the gene in which the mutation occurs or mark the mutation as being in an untranslated region. (A) SINV populations evolved in sudden host‐change treatment. (B) SINV populations evolved in gradual host‐change treatment.

Of the 288 libraries sequenced (18 populations × 8 time points × 2 replicates), 286 produced high‐quality data, and two libraries produced data too poor to be considered for further analysis. In the retained libraries the average depth of coverage was 6127×. For each sample, we recorded the frequency of all genetic variants above a 1% frequency threshold. This allowed us to track the frequency of mutations relative to the ancestral genome over time (Fig. 1). Of mutations recorded above the 1% threshold, 94.86% were single‐nucleotide polymorphisms (SNPs) and 5.14% were indels. A total of 45.46% of the SNPs were nonsynonymous mutations and 49.39% were synonymous. Of the mutations that reached fixation, all of which were single nucleotide changes, 78.13% were nonsynonymous and 21.87% were synonymous. We tracked an average of 192 mutations per population. Three hundred ninety of the mutations we observed were detected in multiple populations. Of this subset, 252 mutations were observed in both treatments, 67 were observed multiple times but only in the sudden treatment, and 71 were observed multiple times but only in the gradual treatment.

### RELATIONSHIP BETWEEN MUTATIONAL EFFECT SIZE AND FIXATION TIMING DEPENDS ON RATE OF HOST CHANGE

**Figure 2:**
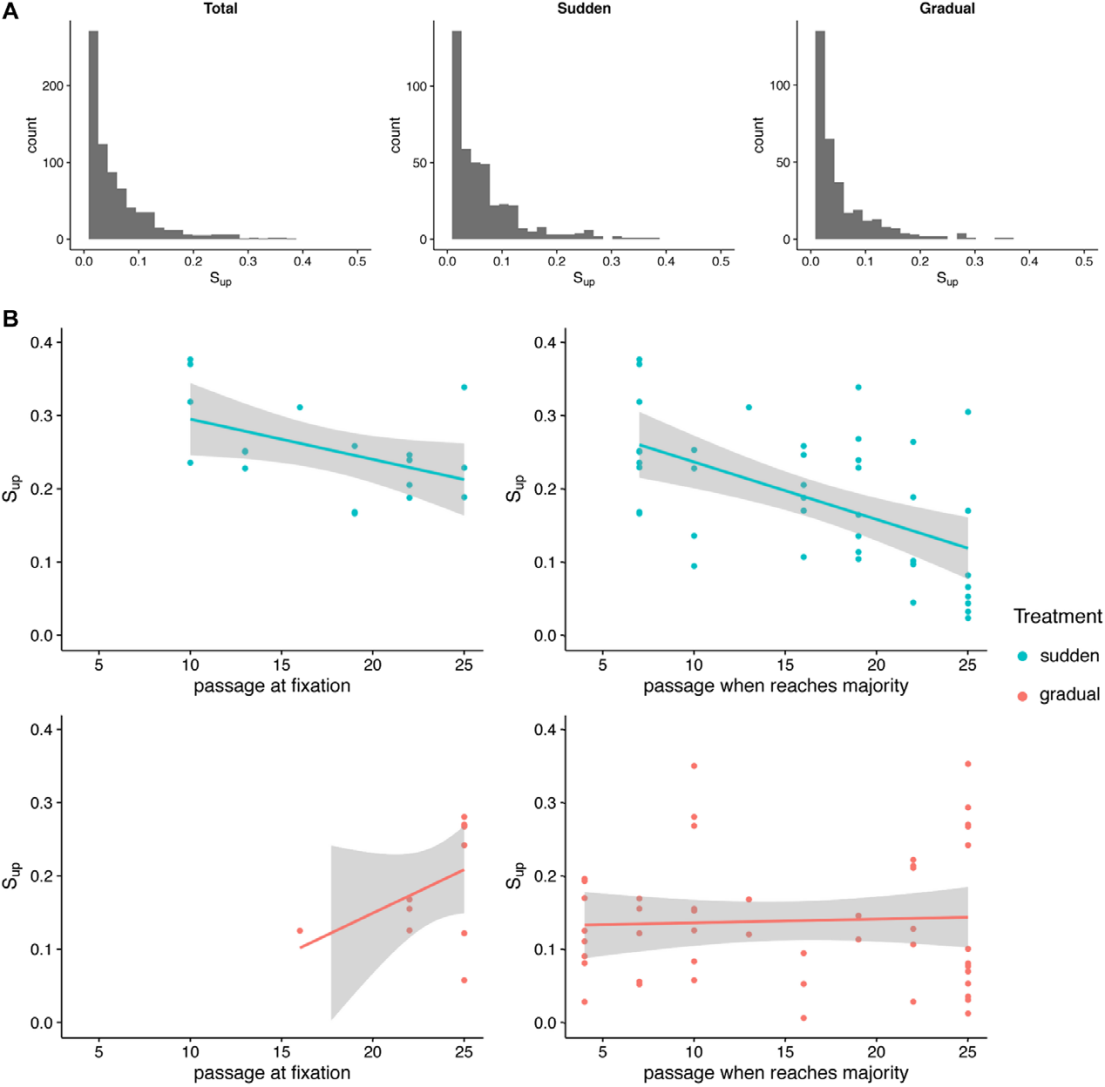
Distribution and correlation with fixation times for *S_up_* values. (A) Histograms showing the distribution of *S_up_* values for the total set of mutations for which *S_up_* was calculated and for those in each treatment. (B) Mutational *S_up_* values versus the passage at which that mutation reaches fixation or majority. Upper panels (blue) show relationships for sudden treatment, lower panels (pink) show relationships for gradual treatment.

To analyze the relationship between fixation time and mutational effect size, we calculated *S_up_*, an estimate of the relative fitness of a mutation and its genetic background at the time of first detection (Lang et al. 2011, 2013). Overall, we found that the distribution of beneficial mutational effect sizes was roughly exponential, with the majority of beneficial mutations conferring a small fitness effect, and fewer mutations conferring large beneficial effects (Fig. 2A). We next considered two subsets of mutations: mutations that reached fixation (frequency = 100%) and the larger group of mutations that reached the majority (frequency > 50%). For mutations that reached fixation in the sudden treatment, we found a significant correlation between *S_up_* and timing of fixation (Pearson correlation, *t* = −2.194, df = 16, *P*‐value = 0.043; Fig. 2B). Mutations with higher *S_up_* and thus higher beneficial effects tended to fix earlier in the experiment. A similar pattern was observed for mutations reaching majority (*t* = −4.0562, df = 39, *P*‐value < 0.001). In the gradual treatment, these patterns disappeared. There was no correlation between *S_up_* value and the time at which mutations fixed (*t* = 1.405, df = 9, *P*‐value = 0.193) or reached the majority (*t* = 0.903, df = 46, *P*‐value = 0.371). The overall pattern and significance of these results were not affected by pooling data for mutations that appeared to be on the same genetic background (i.e., treating multiple mutations that appear and rise in frequency together as a single mutation; Fig. S3). These data support our first hypothesis that the relationship between mutational effect size and order of fixation differs based on the rate of environmental change.

### PARALLELISM IN EARLY MUTATIONAL STEPS

Based on the observed correlation between mutational effect size and time of fixation, we expected there to be stronger parallelism in early mutational steps in the sudden treatment. In fact, we observed that early mutational steps were highly parallel across replicate populations in both treatments (Fig. 1). In the sudden treatment, five of the nine populations had the same mutation (A11121G) as the first to sweep to fixation. This mutation causes an amino acid substitution in the ectodomain of the E1 protein (T353A), which is involved in the fusion of viral and cellular membranes (Kuhn 2013). In each of the remaining four populations, the first mutation to sweep to fixation was nonsynonymous and occurred within amino acid residues 44–181 of the E2 protein, which is involved in viral attachment to the host receptor (Kuhn 2013). We note that experimental populations were founded from an infectious clone amplified for a single passage on BHK cells; therefore, we cannot rule out the possibility that these mutations could have been present at low levels in the initial inoculum.

In the gradual treatment, the most convergent pattern was the synchronous early rise and then fall of a pair of mutations: A3448T and A9109T. A3448T causes an amino acid substitution in the protease region of the nsP2 protein (K590M), and A9109T causes an amino acid substitution in the ectodomain of the E2 protein (E160V). The early rise and fall of this pair of mutations was observed in six of nine replicate populations.

### DYNAMICS DOMINATED BY SWEEPS OF MULTIPLE ASSOCIATED MUTATIONS

**Figure 3:**
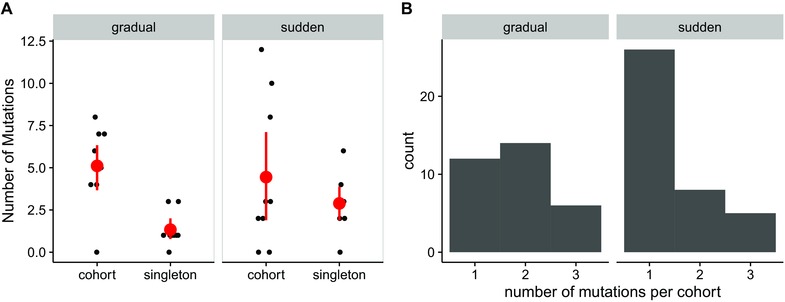
Analysis of mutational cohorts. (A) Counts of mutations classified as singletons and mutations classified as members of cohorts for each virus population. Each point represents the number of singletons or mutations in cohorts for a single virus population. Means and 95% CI are shown in red. (B) The distribution of cohort sizes by treatment. Here, singletons are considered cohorts of one.

To test our second hypothesis, we looked for evidence that groups of mutations changed in frequency synchronously with each other over time. This pattern is expected if mutations arise on the same genetic background. We used hierarchical clustering of frequency trajectories to empirically cluster associated mutations into cohorts; otherwise we labeled them as singletons (Materials and Methods). Of mutations that at some point reached 30% frequency in our experiment, we found that 70% traveled as members of mutational cohorts rather than as singletons. The bias toward cohorts was greater in the gradual treatment than in the sudden treatment (Fig. 3A). In the gradual treatment, there were significantly more mutations classified as members of cohorts than as singletons (two‐sided *t*‐test, *t* = 4.408, df = 10.764, *P*‐value = 0.001). In the sudden treatment, there was no significant difference between the number of singletons versus cohort members (*t* = 0.991, df = 10.102, *P*‐value = 0.344).

We also analyzed the distribution of mutational cohort sizes (Fig. 3B), here considering singletons to be cohorts with only one member. We found that cohort size depended significantly on treatment, with cohort size skewing larger in the gradual treatment (general linear model, *P* = 0.047). This result means that genomes reaching frequencies >30% in the gradual treatment generally carry more mutations than do those in the sudden treatment.

A surprising result from our experiment was the repeated observation of the same mutational cohorts across multiple replicate populations. Three cohorts were observed multiple times, and these cohorts exclusively occurred in the gradual treatment. The combination T8698C (E2 V23A) + A11447G (3’UTR) was observed in two populations, A7806G (C I53V) + T8698G (E2 V23G) was observed in three populations, and A3448T (nsP2 K590M) + A9109T (E2 E160V) was observed in six populations. Mutations T8698C, A11447G, A3448T, and A9109T were never observed above the 30% threshold outside of these specific cohorts.

### CLONAL INTERFERENCE APPEARS STRONGER WITH GRADUAL CHANGE

Under clonal interference, beneficial mutations can be lost as a result of failed competition with other beneficial mutations. Therefore, when clonal interference is stronger, there is an increase in mutations that rise to appreciable frequencies, but then are subsequently lost. The probability that mutations that reach frequency *x* are subsequently lost can be described by the parameter *h(x)* (Strelkowa and Lässig 2012)*. h(x)* is defined as follows:

$$h\left(x\right)=\frac{H{\left(x\right)}_{nonsynonymous}}{H{\left(x\right)}_{synonymous}},$$

where *H(x)_nonsynonymous_* is the number of nonsynonymous mutations that reach frequency *x* and are subsequently lost, and *H(x)_synonymous_* is that for synonymous mutations. If we assume that synonymous mutations are likely to be neutral while nonsynonymous mutations are more likely to have fitness effects, *h(x)* > 1 suggests that mutations are rising in frequency due to positive selection and later being lost. This is in indication of clonal interference. Not all synonymous changes are neutral in ssRNA viruses (Olsthoorn and van Duin 1996; Klovins et al. 1997a,1997b; Yoshida et al. 1997; Simmonds and Smith 1999; Cuevas et al. 2012; Chen et al. 2013); however, nonlethal synonymous mutations have a distribution of fitness effects centered near neutrality (Cuevas et al. 2012; Acevedo et al. 2014). While positively selected synonymous mutations could feasibly dampen the signal in this analysis, synonymous mutations generally still serve as a useful baseline for comparison.

**Figure 4:**
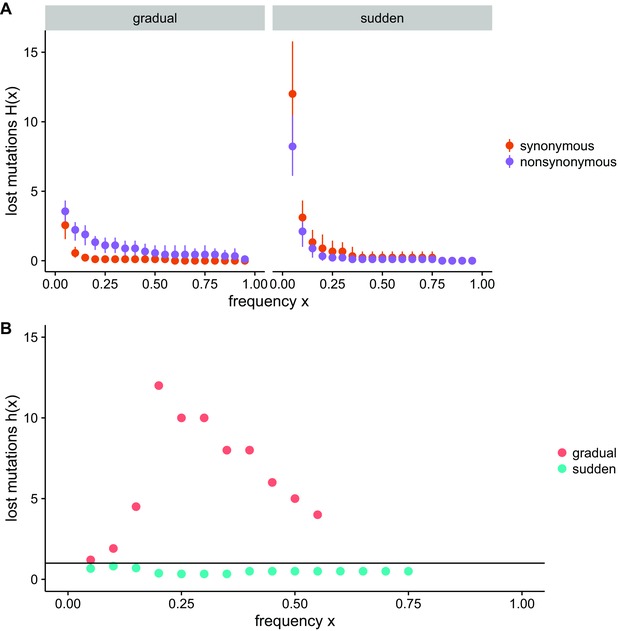
Likelihood of mutational loss. (A) Likelihood of loss *H(x)* after reaching frequency *x* for nonsynonymous and synonymous mutations. (B) A summary ratio *h(x)* calculated over the whole dataset. When *h(x)* > 1 (threshold marked by line), nonsynonymous mutations are lost at a higher rate than synonymous mutations.

Due to the timescale of our experiment, in multiple populations at least one component of the *h(x)* ratio was equal to 0, so we were not able to calculate *h(x)* for each population. However, we calculated *H(x)_nonsynonymous_* and *H(x)_synonymous_* across all experimental populations (Fig. 4A). In the gradual treatment frequency *x*, mutation type (synonymous or nonsynonymous), and an interaction term are all highly significant predictors of *H(x)* (GLM, frequency: *P* < 0.001 , type: *P* < 0.001, frequency × type: *P* < 0.001). Nonsynonymous mutations have a significantly higher *H(x)* than synonymous mutations. In the sudden treatment, the type of mutation had a less significant effect, and the effect was in the opposite direction (GLM, frequency: *P* < 0.001, type: *P* = 0.012, frequency × type: *P* = 0.047). In the sudden treatment nonsynonymous mutations had significantly lower *H(x)* than synonymous mutations. Although we could not calculate *h(x)* for individual populations, we calculated the ratio *h(x)* using all mutations in the dataset to provide a sense of the dynamic. These data indicated that positively selected mutations reach intermediate frequencies only to be subsequently lost more often in the gradual treatment. This strongly suggests a larger effect of clonal interference in response to a gradually changing environment.

## Discussion

Our results support theoretical predictions that the rate of environmental turnover should affect the dynamics of adaptation (Waxman and Peck 1999; Collins et al. 2007; Kopp and Hermisson 2009a,2009b; Schiffels et al. 2011). Most strikingly, our results confirm that mutations of large beneficial effect tend to fix early when the environment changes suddenly; however, when the environment changes gradually the correlation between mutational effect size and time of fixation is lost. These results align closely with previous experimental work conducted with the unicellular alga *Chlamydomonas reinhardtii* (Collins and de Meaux 2009). *Chlamydomonas* populations were exposed to a sudden or gradual increase in phosphate concentration, and mutational sweeps were described based on fluctuations in the frequencies of marked lineages. The authors found that sudden environmental change resulted in large increases in fitness early in the experiment, but gradual change resulted in populations that adapted through smaller increases in fitness that occurred at less predictable times. Our work expands these results to RNA viruses, a model with very different genomic architecture, suggesting that these dynamics may be common across asexual systems. Additionally, the small genomes of RNA viruses allowed us to track mutational frequency through whole‐genome sequencing, illuminating molecular dynamics at a higher resolution than had been previously achieved.

When the same mutation is observed in multiple populations, there can be variation in the *S_up_* values. This can occur for two reasons that are not mutually exclusive. First, variation in *S_up_* values can indicate epistasis. The *S_up_* parameter describes the fitness of a mutation and the genetic background in which it occurs, so variation across populations could indicate an effect of genetic background. Second, differences in *S_up_* values could reflect differences in the fitness of the population at the time the mutation occurs. *S_up_* measures the fitness of a genome relative to the mean fitness of the population at the time point when the mutation is first detected. Relatedly, the *S_up_* values of mutations could be affected by the strength of clonal interference. If clonal interference affects the rate at which a mutation's frequency changes after first detection, this would affect *S_up_* estimates. We predict that this would most affect *S_up_* values for weakly selected mutations that would have longer wait times with stronger CI; however, the greatest effect here may occur when mutations are still below our threshold of detection.

Our results revealed viral adaptation characterized by sweeps of mutational cohorts. Mutations may sweep through the population synchronously as cohorts for several reasons. One is genetic hitchhiking, wherein a beneficial mutation sweeps to fixation and carries along any other neutral or mildly deleterious mutations occurring on the same genetic background. Alternatively, two independent beneficial mutations may happen to occur on the same genetic background and sweep to fixation together. Finally, some cohorts may be favored due to epistatic interactions; if two mutations are beneficial in combination, this could drive genomes containing both mutations to rise in frequency. In the current experiment, we were surprised to observe that some cohorts of mutations appeared convergently across multiple populations. This convergence is unlikely to result from hitchhiking, but could be the result of epistasis or independently beneficial mutations co‐occurring.

The cohort A3448T + A9109T was observed rising and then declining early in the adaptive walks of six populations in the gradual treatment. In our previous study all 144 SINV populations were sequenced via Sanger sequencing at the endpoint of the experiment, and this larger sample size may provide some insight into the role of these mutations (Morley et al. 2015). Previously, the mutation A3448T was detected in two populations evolved strictly on BHK cells, while the mutation A9109T was observed only in populations that transitioned onto CHO cells either suddenly or gradually (9/124 populations). If A3448T is beneficial on BHK cells but deleterious on CHO cells, the changing selection coefficient could explain the observed pattern. However, reason for the parallel co‐occurrence of these two mutations in the gradual treatment remains to be determined.

Future work could genetically engineer viruses with mutations singly or in combination to further investigate the role of epistasis in shaping adaptive walks; however, this is beyond the scope of the current study. The current study and others like it identify a large number of mutations, and the number of possible combinations becomes challenging to engineer. Other studies have shown that epistatic interactions, including those involving more than two loci, are likely to play a key role in virus evolution (Sanjuan et al. 2004, 2005; Pepin and Wichman 2007; da Silva et al. 2010; Rokyta et al. 2011; Caudle et al. 2014; Parera and Martinez 2014). Further integrating epistasis into our understanding of evolutionary dynamics and clonal interference remains a crucial direction for future study.

Experimental studies of adaptation have typically focused on sudden environmental changes. While some environmental changes in nature do happen suddenly, environmental changes are often more gradual. Several recent studies have explored microbial evolution in response to gradual changes in the abiotic environment, such as gradually increasing antibiotic concentrations (Collins and de Meaux 2009; Lindsey et al. 2013; Gorter et al. 2016). However, the biotic environment can also turn over at variable rates. Here, we offer two examples to illustrate instances when a virus population might experience gradual turnover in its host community.

First, gradual turnover of hosts can happen at the cellular level, here illustrated by human immunodeficiency virus (HIV) infections. To successfully infect a target cell, HIV must bind to the primary cell surface molecule CD4 and also to a secondary receptor. The most important secondary receptors are CCR5 and CXCR4 (Berger et al. 1999). These two receptors are primarily expressed on different types of cells: CCR5 is more often expressed in memory T cells and CXCR4 is more often expressed in naïve T cells (Bleul et al. 1997; Lee et al. 1999). Over the course of infection, the relative prevalence of these two types of cells in the host changes, notably influenced by an increase in proliferation rate of CXCR4‐expressing naïve T cells (Regoes and Bonhoeffer 2005). This change in the composition of the CD4+ T cell population has been proposed as an explanation for a commonly observed pattern in HIV progression. Virus lineages specific to CCR5 cells dominate early HIV infections, but in about half of all cases, virus lineages specific to CXCR4 cells dominate late infections. One hypothesis is that this shift in host specificity is selected for due to the gradual shift in availability of the two target cell types (Davenport et al. 2002; Regoes and Bonhoeffer 2005; Ribeiro et al. 2006).

Second, gradual turnover of hosts can happen at the level of multi‐species communities. Here, we focus on the example of the invasive mosquito *Aedes albopictus*. *Ae. albopictus* is native to Asia, but in recent decades it has invaded tropical and subtropical habitats around the globe facilitated by the shipment of used tires (Lounibos 2002). Frequently, the regions *Ae. albopictus* invaded were already populated by the mosquito *Aedes aegypti*, itself a previous invader. In some regions declines in *Ae. aegypti* populations were documented in response to *Ae. albopictus* invasion (O'Meara et al. 1995; Lounibos 2002; Juliano and Lounibos 2005). Importantly, both of these mosquito species are key vectors for arboviruses such as dengue virus and chikungunya virus. A gradual change in the vertebrate host and/or the invertebrate vector community composition resulting from an invasion is likely to have evolutionary consequences for viral populations. This is because an arbovirus lineage can experience selection to improve infection of the vertebrate host, as well as selection to enhance replication within the invertebrate vector, such as increased virus reproduction in the mosquito midgut and travel to the mosquito salivary gland to foster transmission. For chikungunya virus, evolutionary pressure to adapt to *Ae. albopictus* lead to multiple convergent emergences of lineages with an alanine residue at position 226 of the E1 gene, which confers higher fitness on *Ae. albopictus* (Schuffenecker et al. 2006; Tsetsarkin et al. 2007; de Lamballerie et al. 2008). Invasions such as this one can create situations where a virus is increasingly likely to encounter a novel host as time progresses, resulting in selective pressures for tropism that shift over time.

We anticipate that next‐generation sequencing technologies combined with experimental microbial evolution will continue to be a powerful method to test evolutionary theory at the molecular level. In particular, experimental evolution is a useful first step in untangling the relationship between environmental complexity and the dynamics of adaptation. Experiments in microbial evolution serve as a tractable bridge between adaptive theory and understanding adaptation in the spatially and temporally heterogeneous environments of the natural world.

Associate Editor: T. Cooper

Handling Editor: M. Noor

## Supporting information


**Figure S1**. Estimation of error in allele frequency measurements.
**Figure S2**. The percentage of CHO cells in the environment at each experimental passage.
**Figure S3**. Correlation with fixation times for *S_up_* values with cohorts grouped.
