# Supplementary material for: Dynamics of molecular evolution in RNA virus populations depend on sudden versus gradual environmental change
Source: Evolution. 2017 Feb 14;71(4):872–83. doi: 10.1111/evo.13193 (PMC5382103; doi:10.1111/evo.13193)
Supplement: Supplementary file 1 — Figure S1. Estimation of error in allele frequency measurements. Figure S2. The percentage of CHO cells in the environment at each experimental passage. Figure S3. Correlation with fixation times for Sup values with cohorts grouped. [file EVO-71-872-s001.docx]

**Supplementary Figure 1**

**Estimation of error in allele frequency measurements.**  Two technical replicates were amplified and sequenced for each biological sample. Each reported allele frequency is the mean of two replicate measurements. To estimate the error in our measurements, we calculated the difference between each measurement and the reported mean. The distribution of these differences (as absolute values) is shown here. The mean error was 0.0026 with a standard deviation of 0.0048.

**Supplementary Figure 2**

The percentage of CHO cells in the environment at each experimental passage. Triangles represent the sudden treatment, and squares represent the gradual treatment.

**Supplementary Figure 3**

Correlation with fixation times for *S_up_* values with cohorts grouped. An additional analysis of the data presented in Figure 2 with the data set edited to consider cohorts of mutations with the same *t_1_* as single mutations. Mutational *S_up_* values versus the passage at which that mutation reaches fixation or majority. Upper panels (blue) show relationships for sudden treatment, lower panels (pink) show relationships for gradual treatment.

For mutations that reached fixation in the sudden treatment, we found a significant correlation between *S_up_* and timing of fixation (Pearson correlation, t = −2.865, df = 20, p-value = 0.010). A similar pattern was observed for mutations reaching majority (t = −4.003, df = 46, p-value = < 0.001). In the gradual treatment, there was no correlation between *S_up_* value and the time at which mutations fixed (t = 1.745, df = 11, p-value = 0.102) or reached the majority (t = 1.057, df = 56, p-value = 0.295).
